# Combined treatment with HMGN1 and anti-CD4 depleting antibody reverses T cell exhaustion and exerts robust anti-tumor effects in mice

**DOI:** 10.1186/s40425-019-0503-6

**Published:** 2019-01-29

**Authors:** Chang-Yu Chen, Satoshi Ueha, Yoshiro Ishiwata, Shoji Yokochi, De Yang, Joost J. Oppenheim, Haru Ogiwara, Shigeyuki Shichino, Shungo Deshimaru, Francis H. W. Shand, Shiro Shibayama, Kouji Matsushima

**Affiliations:** 1 Division of Molecular Regulation of Inflammatory and Immune Diseases, Research Institute for Biomedical Sciences, Tokyo University of Science, Chiba, Japan; 2 Department of Molecular Preventive Medicine, Graduate School of Medicine, Tokyo University of Science, 2669 Yamazaki, Noda, Chiba, 278-0022 Japan; 3 Cancer and Inflammation Program, Center for Cancer Research, National Cancer Institute at Frederick, Frederick, MD USA; 4 Research Center of Immunology, Tsukuba Institute, ONO Pharmaceutical Co., Ltd., Tsukuba, Japan

**Keywords:** High mobility group nucleosome binding protein 1 (HMGN1), Anti-CD4 depleting antibody, Combination cancer immunotherapy

## Abstract

**Background:**

Transient depletion of CD4^+^ T cells results in tumor suppression and survival benefit in murine models; however, the tumor progression and recurrence still occur over more long-term monitoring of mice. Thus, we explored an additional strategy to enhance endogenous immune responses by an alarmin, high mobility group nucleosome binding protein 1 (HMGN1).

**Methods:**

The anti-tumor effects of HMGN1, anti-CD4 depleting antibody, and their combined treatment were monitored in the Colon26 or the B16F10 subcutaneous murine models. The tumor-infiltrating CD8^+^ T cell proliferation, differentiation, exhaustion, and its gene expression were determined by flow cytometry, transcriptome analysis, and quantitative real-time PCR.

**Results:**

Our results show that a systemic administration of low doses of HMGN1 with an anti-CD4 depleting antibody (HMGN1/αCD4) promoted expansion of CD8^+^ T cell populations (e.g. CD137^+^ PD-1^+^ and CD44^hi^ PD-1^+^), recruited CCR7^+^ migratory dendritic cells to the tumor, and reduced co-inhibitory molecules (e.g. PD-1, LAG-3, and TIM-3) to counteract CD8^+^ T cell exhaustion.

**Conclusion:**

The HMGN1/αCD4 treatment expanded effector CD8^+^ T cells and prolonged their anti-tumor activities by rescuing them from exhaustion, thus resulting in tumor regression and even rejection in long-term monitored mice.

**Electronic supplementary material:**

The online version of this article (10.1186/s40425-019-0503-6) contains supplementary material, which is available to authorized users.

## Background

Immunosuppression induced by cancer cells and CD4^+^ immunosuppressive cells (e.g. Foxp3^+^ T regulatory cells) limits the anti-tumor effect of effector CD8^+^ T cells [1–3]. Among the strategies used to reverse immunosuppression, cancer immunotherapy that targets immune checkpoints such as cytotoxic T-lymphocyte-associated antigen-4 (CTLA-4), programmed cell death 1 (PD-1) or programmed cell death ligand 1 (PD-L1) of immune checkpoint has demonstrated remarkable efficacy in various cancers. Transient depletion of CD4^+^ immunosuppressive cells in the tumor-bearing host represents another potential anti-tumor therapeutic strategy against cancer. Previously we have reported that in tumor-bearing mice, CD4 depletion with an anti-CD4 depleting antibody (clone: GK5.1) enhances the proliferation of effector CD8^+^ T cells in the draining lymph node and results in potential anti-tumor immunity [2]. Furthermore, co-administration of anti-CD4 depleting antibody and anti-PD-1 or anti-PD-L1 antibodies resulted in rejection of established tumors in some mice [2]. However, these combination therapies are not effective in all individuals, highlighting the need for additional anti-tumor strategies, such as enhancement of endogenous immune responses.

Here we focus on high mobility group nucleosome binding protein 1 (HMGN1), an alarmin and endogenous damage-associated molecular pattern (DAMP) molecule that is released from cells via the endoplasmic reticulum-Golgi secretion pathway or via non-programmed cell death after cellular stress or damage [4, 5]. HMGN1 serves as a potent immune stimulator that promotes dendritic cell (DC) activation and migration, and consequently polarizes Type 1 T helper cell-mediated immune responses, which enhance anti-tumor immunity in mice [6–9].

In 2017, a new cancer vaccine consisting of HMGN1, toll-like receptor agonist (e.g. R848) and immune checkpoint blockade (e.g. anti-PD-L1 or anti-CTLA-4 antibody) was termed TheraVac. Intratumoral administration of TheraVac in mice promotes DC activation and CD107α^+^ CD8^+^ T cell expansion in the tumor and results in inhibition of tumor growth [10, 11]. These finding suggest that extracellular HMGN1 triggers local anti-tumor responses through intratumoral administration. However, it remains to be established whether systemic administration of HMGN1 also influences anti-tumor T cell responses.

In this study, we evaluated the anti-tumor effects and immunological responses induced by co-administration of HMGN1 and anti-CD4 depleting antibody intraperitoneally in Colon26 or B16F10 tumor-bearing mice. We also investigated the effect of HMGN1 on the gene expression of CD8^+^ T cells. Our results demonstrate that the combined treatment with low-dose HMGN1 and anti-CD4 depleting antibody exerts synergistic anti-tumor effects by promoting CD8^+^ T cell activation, expansion, and by counteracting exhaustion of activated CD8^+^ T cells in the tumor.

## Methods

### Mice

Seven-week-old female BALB/c, BALB/c-nu, and C57BL/6 mice were purchased from Japan Charles River. Pmel-1 (B6.Cg-Thy1a/Cy Tg(TcraTcrb)8Rest/J) mice with transgenic gp100 melanoma antigen-specified T cell receptor and Ly5.1 (B6.SJL-Ptprca Pepcb/BoyJ) mice were purchased from the Jackson Laboratory (ME, USA). Each experiment group contained 8 mice except where otherwise specified. All animal experiments were conducted in accordance with institutional guidelines with the approval of the Animal Care and Use Committee of the University of Tokyo and the Tokyo University of Science.

### Cell lines and tumor models

Colon26 cells were obtained from the Cell Resource Center for Biomedical Research (RRID: CVCL_0240; Institute of Development, Aging, and Cancer, Tohoku University, Japan). B16F10 cells were obtained from the American Type Culture Collection (RRID: CVCL_0159; ATCC, USA). Colon26 cells (2 × 10^5^ cells / mouse) and B16F10 cells (5 × 10^5^ cells / mouse) were inoculated subcutaneously into the right flank of BALB/c, BALB/c-nu or C57BL/6 mice. Tumor diameter was measured by twice weekly and used to calculate tumor volume (V, mm^3^) using the formula V = L × W × W/ 2 (where L is tumor length and W is tumor width).

### Recombinant HMGN1

Recombinant HMGN1 proteins were produced in *E. coli* and purified using sequential fractionation by heparin affinity column, ion exchange column and reverse-phase column. The final protein products had over 99% purity with under 1 endotoxin unit concentration per microgram protein, as assessed by SDS-PAGE and an Endospecy ES-50 M Kit (Seikagaku Corporation, Japan), respectively. Detail of the production and purification of mouse and human recombinant HMGN1 proteins is described in Additional file 1: Method S1, Figure S1 and Table S1.

### In vivo treatment

HMGN1 protein (at a dose of 0.16 μg per mouse per injection, unless otherwise specified) was administered intraperitoneally on days 9, 14, 17, and 20 after tumor inoculation. Anti-CD4 depleting antibody (clone GK1.5; BioXcell, USA) was injected intraperitoneally on days 5 and 9 after tumor inoculation, at a dose of 200 μg per mouse per injection [2]. The optimized protocol for B16F10 tumor-bearing mice is described in Additional file 1: Figure S2.

### Flow cytometry and cell sorting

Three minutes before collecting tissues, intravascular leukocytes were stained by intravenous injection of fluorescein isothiocyanate (FITC)-conjugated antibody (3 μg/mouse) against CD45 [12]. Single cell suspensions were prepared by enzymatic or mechanical dissociation of tissues with or without subsequent density separation, as described previously [13, 14]. Flow-Count fluorospheres (Beckman Coulter, USA) were used to determine cell numbers. Cells were pretreated with Fc block reagents (anti-mouse CD16/CD32 antibody, clone 2.4G2; BioXcell), then stained with a mix of fluorophore-conjugated anti-mouse antibodies as indicated in Additional file 1: Table S2. Data were acquired on a Gallios flow cytometer (Beckman Coulter) and analyzed by using FlowJo 10.5.3 software (FlowJo, LLC, USA). Nonviable cells were excluded from the analysis based on forward and side scatter profiles, and dead cells were excluded by propidium iodide (PI) staining. For intracellular cytokine detection, enriched tumor-infiltrating CD8^+^ T cells were re-stimulated with 1 μg/ml ionomycin (IM) and 25 ng/ml phorbol myristate acetate (PMA) in the presence of GolgiPlug (BD Biosciences, USA) for 4 h at 37 °C. The re-stimulated CD8^+^ T cells were stained with surface antigens, and these cells were stained for intracellular cytokines using a Cytofix/Cytoperm kit (BD Biosciences, USA), according to the manufacturer’s instructions. For the transcriptome analysis, CD8^+^ T cells from the tumor were sorted on FACSAria II Cell Sorter (BD Biosciences, USA).

### Murine BMDC generation and treatment

Bone marrow cells were extracted from the femurs of Ly5.1 mice and hematopoietic progenitors were enriched by depleting lineage (CD3, B220, NK1.1, Ly-6G, Ter119) positive cells with magnetic beads (Miltenyi Biotec, Germany). Bone marrow-derived dendritic cells (BMDCs) were generated by culturing hematopoietic progenitors for 7 days in complete medium (RPMI 1640, 55 μM 2-mercaptoethanol, 1 mM sodium pyruvate, 10 mM HEPES, 100 U/mL Penicillin-Streptomycin, 0.1 mM non-essential amino acids, and 10% fetal bovine serum) with 20 ng/mL GM-CSF. After 7-days of culture, immature BMDCs were further cultured in maturation medium (complete medium with 10 ng/mL GM-CSF and 0.5 μg/mL lipopolysaccharide) for 24 h.

### Ex vivo CD8 T cell expansion assay

Pmel-1 (CD90.1^+^) CD8^+^ T cells were enriched from spleen single cell suspensions by depleting the lineage (CD4^+^, CD11b^+^, CD11c^+^, B220^+^, NK1.1^+^, Ter119^+^) on an autoMACS cell separator (Miltenyi Biotec, Germany). Pmel-1 CD8^+^ T cells were labeled with carboxyfluorescein diacetate succinimidyl ester (CFSE) at a final concentration of 2 μM/ 3 × 10^6^ cells/ml for 5 min at room temperature. In the DC-dependent assay, CFSE-labeled Pmel-1 CD8^+^ T cells were cultured with gp100-pulsed BMDCs (pre-stimulation with 1 μg/mL gp100 for 2 h) in complete medium with or without 100 ng/mL HMGN1 for 48 h. In the DC-independent assay, CFSE-labeled Pmel-1 CD8^+^ T cells were cultured in a dish pre-coated with anti-CD3/CD28 antibodies with complete medium with or without 100 ng/mL HMGN1 for 72 h. The proliferation of activated Pmel-1 CD8^+^ T cells (CD25^+^CD90.1^+^CD8^+^) was assessed by CFSE intensity using flow cytometry.

### Transcriptome analysis

The whole transcripts were amplified from sorted CD8^+^ T cells and those transcripts were used to generate the 3’end Serial Analysis of Gene Expression (SAGE)-sequencing libraries (Additional file 1: Method S2). The sequencing was performed by using an Ion Hi-Q Chef kit, an Ion PI v3 Chip kit, and an Ion Proton Sequencer (Thermo Fisher Scientific) according to the manufacturer’s instructions except the input library concentration was 100 pM. Adapter trimming and quality filtering of sequencing data were performed by using Trimommatic-v0.36 [15] and PRINSEQ 0.20.4 [16]. The filtered reads were mapped on Refseq mm10 using Bowtie2–2.2.5 (parameters: -t -p 11 -N 1 -D 200 -R 20 -L 20 -i S,1,0.50 --norc). The reads unmapped to NlaIII cutting sites were removed, and the mapped reads per gene (raw tag counts) were be quantified as gene expression. Between-sample normalization of gene expression was performed against raw count data by using R 3.4.3 (https://cran.r-project.org/) with TCC [17], DESeq2 [18], and edgeR [19] packages. Genes with adjusted *p* value less than 0.05 and a fold-change of ≥ 2 between at least two samples were identified as statistically significant differentially expressed genes (DEGs). Raw data from the experiment have been deposited in the NCBI Gene Expression Omnibus (GEO, http://www.ncbi.nlm.nih.gov/geo); accession GSE113307.

### Functional annotation of DEGs

Functional analysis of DEGs was performed by using Cytoscape 3.6.0 with ClueGO plugin (v2.5.0) [20, 21]. Significantly enriched Gene Ontology (GO) terms [22] (GO-biological process, GO levels: 3–8) and Kyoto Encyclopedia of Genes and Genomes (KEGG) pathway terms [23] in DEGs were explored and grouped, and a term network was constructed based on the overlap of their elements (kappa score = 0.4). Leading terms within each group were defined as the most significantly enriched term in each group. Terms not connected with any other term were excluded. Significantly-enriched functional terms (adjusted *p* values < 0.05) for which genes from up- or down-regulated genes comprised over 60% of all genes are shown in the Additional file 1: Table S4. We used versions of the GO term database (Nov. 20, 2017) and KEGG pathway term database (Nov. 20, 2017).

### Quantitative real-time PCR analysis

The primers used in this study were designed using the University Probe Library Assay Design Center (Roche, Switzerland) and are listed in Additional file 1: Table S3. Quantitative real time PCR (qPCR) was performed using Thunderbird SYBR qPCR Mix (Toyobo, Japan) or Thunderbird Probe qPCR Mix (Toyobo) on an Applied Biosystem® 7500 qPCR system (Thermo Fisher Scientific, USA). Three independent biological replicates for each group and double technical replicates for each biological replicate were analyzed. Target gene expression was normalized to the expression of the internal control gene, *Gapdh*.

### Statistical analysis

Unless otherwise noted, all experiments were performed at least three times. Data are presented as mean ± SEM. The significance of differential expression of genes in TCC-normalized 3′ SAGE-seq data was calculated by using TCC package (glmL RT formula in edgeR package) in R 3.4.2. The significance of GO term enrichment was calculated by using Cytoscape 3.6.0 with ClueGO plugin (v2.5.0). Correction for multiple comparisons was performed using the Benjamini-Hochberg method. For comparisons between groups in the in vivo study, we used one-way ANOVA with the Dunnett post hoc test in GraphPad Prism software 6.0 (GraphPad Software, USA). For comparisons between the means of two variables, we used two-tailed unpaired Student’s *t*-test in GraphPad Prism software 6.0. All statistical analyses were conducted with a significance level of α = 0.05 (*P* < 0.05).

## Results

### HMGN1/αCD4 treatment exerted robust anti-tumor effects in mice

**Fig. 1:**
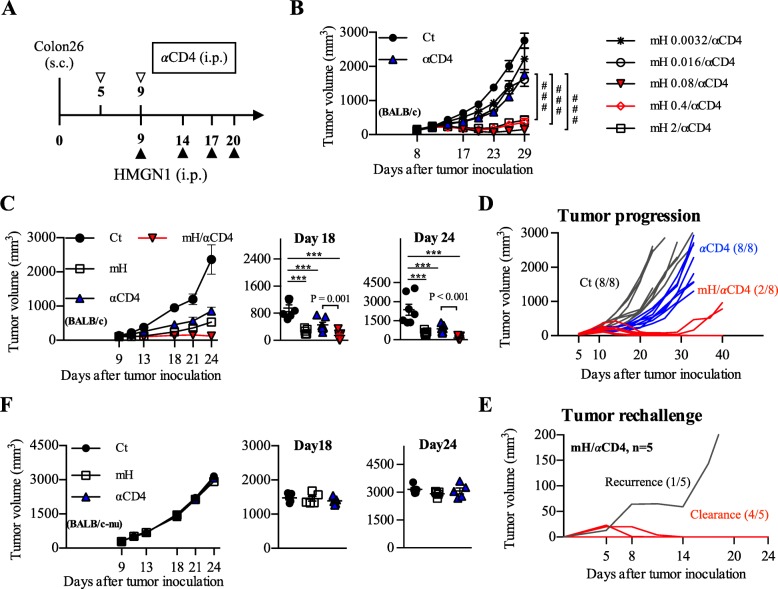
HMGN1/αCD4 treatment exerted robust anti-tumor effects in mice. **a** The optimized protocol for HMGN1/αCD4 treatment in Colon26 model. **b** The effective dose of murine HMGN1 (mH) in combination with anti-CD4 depleting antibody (αCD4) was screened within a range of 0.0032 to 2 μg/mL mH. #, *P* < 0.05, ##, *P* < 0.01, ###, *P* < 0.001 for a Student’s *t*-test comparing mH/αCD4 and αCD4-treated groups at day 29. **c** The tumor growth analysis during mH/αCD4 treatment, and the tumor volume of each mice on day 18. **d** The tumor progression analysis for 40 days. **e** Tumor rechallenge analysis. **f** The tumor growth analysis during mH/αCD4 treatment in Colon26-bearing BALB/c-nu model, and the tumor volume of each mice on day 18. Tumor growth is representative of three independent experiments with at least eight mice per group. Data are presented as mean ± SEM. *, *P* < 0.05, **, *P* < 0.01, ***, *P* < 0.001 for a dunnett’s post hoc test (compared with control); *p* values in the figure indicate Student’s *t*-test comparing mH/αCD4-treated and αCD4-treated groups

Two hundred μg anti-CD4 depleting antibody (αCD4) on day 5 and 9 [2], and various doses (0.0032 to 2 μg/ml) of murine HMGN1 (mH) on day 9, 14, 17, and 20 were administered intraperitoneally to Colon26-bearing mice (Fig. 1a). Monotherapy with αCD4 showed moderate tumor growth inhibition, whereas the combination of αCD4 with mH at doses higher than 0.08 μg per injection showed significant tumor growth inhibition in Colon26-bearing mice (*p* < 0.001) (Fig. 1b). We observed the highest tumor growth inhibition in the mice receiving aCD4 with an mH concentration of 0.08–0.4 μg per injection (Fig. 1b). Based on low doses results, we adopted a dose of 0.16 μg mH per injection as an effective dose in this study. In this setting, combination therapy of mH/αCD4 showed significant tumor growth inhibition relative to monotherapy with αCD4 on day 18 (*p* = 0.001) and day 24 (*p* < 0.001) (Fig. 1c). Similar anti-tumor effects were observed in the combination therapy of αCD4 with human HMGN1 in Colon26-bearing mice (Additional file 1: Figure S2A, B), suggesting that human HMGN1 might share the similar structure and function with murine HMGN1, and might have cross-species activity in its anti-tumor effects.

Moreover, we monitored the long-term outcomes and patterns of tumor progression after treatments. The mice received mH/αCD4 treatment showed low tumor progression (2 out of 8) relative to that of αCD4 treatment (8 out of 8) (Fig. 1d). We re-challenged a quintuple dose (1 × 10^6^) of Colon26 cells in the five cured mice with complete tumor rejection after mH/αCD4 treatment. Four out of five mice resisted to the tumor re-challenge, suggesting that those cured mice might have developed immunological memory (Fig. 1e).

In the case of B16F10 melanoma, mH/αCD4 treatments did not result in significantly smaller tumor volumes relative to αCD4 treatment (Additional file 1: Figure S3A-C). Since the tumor growth of B16F10 model is faster than that of Colon26 model, we gave an additional mH injection to B16F10-bearing mice on day 5 (Additional file 1: Figure S3D). Using this optimized setting, we found that combination with 200 μg αCD4 and 0.08 μg mH significantly reduced tumor volumes compared to monotherapy groups on day 15 (*p* = 0.002) (Additional file 1: Figure S3E, F), suggesting that the proper optimization based on different types of cancers should be considered.

In addition, the anti-tumor effects of mH and αCD4 were not observed in the Colon26-bearing BALB/c-nu mice which lack thymus-dependent T cells, suggesting that anti-tumor effects of these treatments are mediated by CD8^+^ T cell responses (Fig. 1f). Collectively, these results suggest that combination of αCD4 and low-dose HMGN1 (in the range of 0.08 to 0.4 μg per injection), brings T-cell dependent anti-tumor effects in mouse subcutaneous tumor models and has an ability to develop immunological memory against tumor cells.

### HMGN1 expanded CD8^+^ T cells in the tumor

**Fig. 2:**
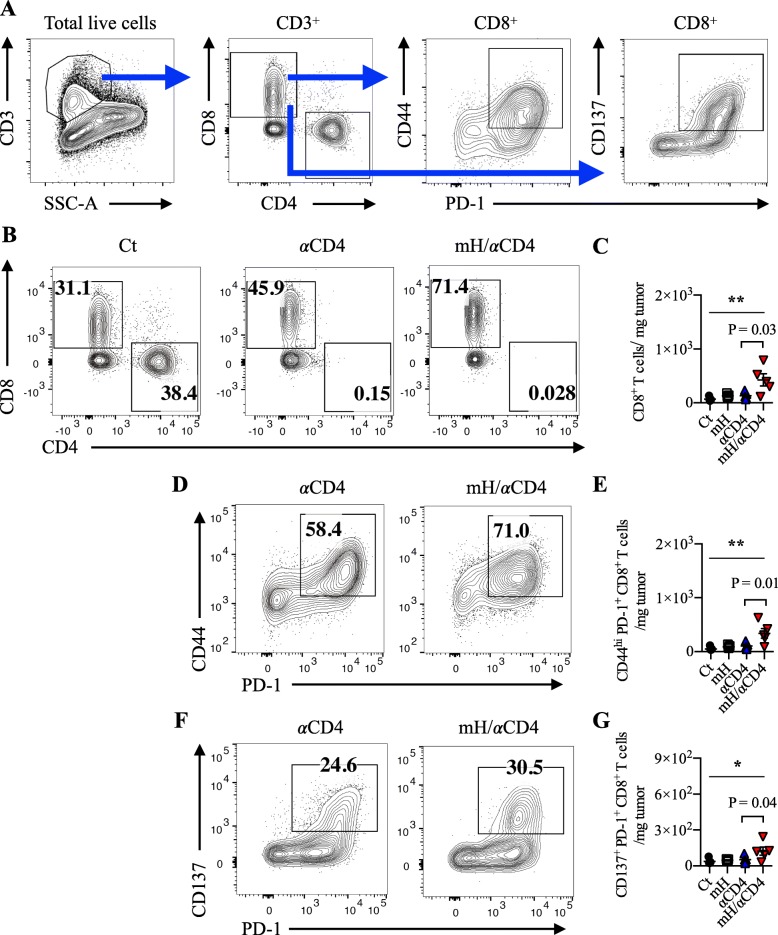
HMGN1 expanded CD8^+^ T cells in the tumor. **a** Flow cytometry gating of tumor-infiltrating CD8^+^ T cell populations. **b**, **c** The compartment, frequency, and cell number of CD8^+^ T cells in the tumor by using flow cytometry. **d**, **e** The compartment, frequency, and cell number of CD44^hi^ PD-1^+^ CD8^+^ T cells in the tumor. **f**, **g** The compartment, frequency, and cell number of CD137^+^ PD-1^+^ CD8^+^ T cells in the tumor. Each result is representative of three independent experiments with at least four mice per group. Data are presented as mean ± SEM. *, *P* < 0.05, **, *P* < 0.01 for a dunnett’s post hoc test (compared with control); *p* values in the figures indicate Student’s *t*-test comparing mH/αCD4-treated and αCD4-treated groups

To investigate the nature of the T-cell mediated anti-tumor immunity during mH/αCD4 treatment, we prepared single cell suspensions from the implanted mouse tumor tissue and draining lymph node on day 13. Among tumor-infiltrating cells, we identified a population of lineage^−^ (CD11b^−^ CD11c^−^ CD49b^−^ Ly-6C^−^ Ly6G^−^ Ter119^−^) CD3^+^ CD4^+^ or CD3^+^ CD8^+^ T cells (Fig. 2a). Most of CD4^+^ T cells were depleted in the tumor and draining lymph nodes after mice were treated with αCD4 (Fig. 2b, Additional file 1: S4A).

The number of CD8^+^ T cells was significantly higher in the αCD4- or mH/αCD4-treated mice than in the untreated control mice (Fig. 2c, Additional file 1: S4B). Although the number of CD8^+^ T cells in the draining lymph node of αCD4- or mH/αCD4-treated mice were equivalent (Additional file 1: Figure S4B), the density of CD8^+^ T cells in the tumor was higher in the mH/αCD4-treated mice than in the αCD4-treated mice (*p* = 0.03) (Fig. 2b, c). Moreover, we also analyzed the subpopulation of tumor-infiltrating CD8^+^ T cells by using flow cytometry (Fig. 2a). The density of tumor-reactive CD137^+^ PD-1^+^ CD8^+^ T cells [24–26] and memory-phenotype CD44^hi^ PD-1^+^ CD8^+^ T cells [27, 28] was significantly higher in mH/αCD4-treated mice than αCD4-treated mice (*p* = 0.03 and *p* = 0.04) (Fig. 2d-g).

Collectively, these results suggested that αCD4 treatment, but not mH treatment, play a major role in the expansion of CD8^+^ T cells in draining lymph node, and that mH treatment, when combined with αCD4 treatment, synergistically increases the number of CD8^+^ T cells in the tumor.

### HMGN1/αCD4 treatment increased CCR7^+ ^CD80^hi ^CD86^hi^ migratory DCs in the tumor

**Fig. 3:**
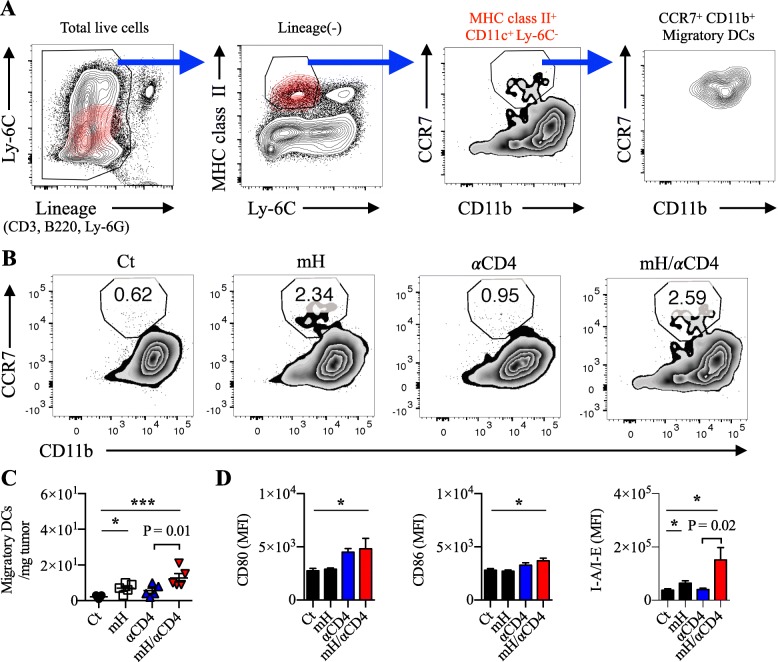
HMGN1/αCD4 treatment increased CCR7^+^CD80^hi^CD86^hi^ migratory DCs in the tumor. **a** Flow cytometry gating of CCR7^+^ migratory DCs. **b**, **c** The frequency and number of CCR7^+^ migratory DCs in the tumor. **d** The expression of CD80, CD86, and I-A/I-E (also known as MHC class II) on CCR7^+^ migratory DCs. Each result is representative of three independent experiments with at least four mice per group. Data are presented as mean ± SEM. *, *P* < 0.05, **, *P* < 0.01, ***, *P* < 0.001 for a dunnett’s post hoc test (compared with control); *p* values in the figure indicate Student’s *t*-test comparing mH/αCD4-treated and αCD4-treated groups. Mean fluorescence intensity (MFI)

To understand why the mH/αCD4 treatment synergistically increases CD8^+^ T cells in the tumor, we focused on the C-C chemokine receptor (CCR) 7^+^ migratory DCs. Previous reports described that CCR7 is essential for the DC trafficking from the tumor to draining lymph nodes. Those CCR7^+^ migratory DCs can support priming and expansion of CD8^+^ T cells [29–31]. Here we defined the CCR7^+^ migratory DCs as lineage^−^ (CD3^−^ B220^−^ Ly6C^−^ Ly6G^−^) MHC class II^+^ CD11c^+^ CD11b^+^ CCR7^+^ cells (Fig. 3a, Additional file 1: S5). On day 13, we found a higher percentage and an increased number of CCR7^+^ migratory DCs in the tumor of mice treated with mH or mH/αCD4 (*p* < 0.05), relative to the mice without mH treatment (Fig. 3b, c). Surprisingly, those increased number of CCR7^+^ migratory DCs with an activated phenotype of CD80^hi^ CD86^hi^ MHC class II^hi^ were only observed in mice received mH/αCD4 treatment (*p* < 0.02) (Fig. 3d), suggesting that those activated CCR7^+^ migratory DCs might drive CD8^+^ T expansion.

### HMGN1 promoted DC-dependent CD8^+^ T cell expansion ex vivo

**Fig. 4:**
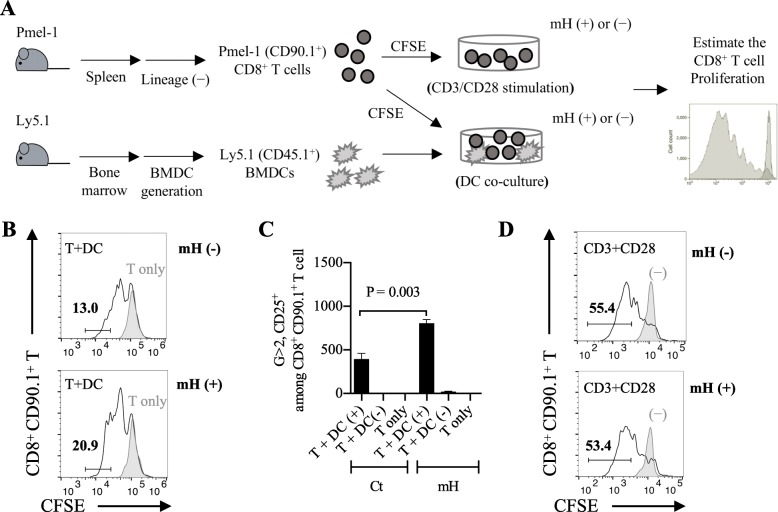
HMGN1 promoted DC-dependent CD8^+^ T cell expansion ex vivo. **a** The experimental design of CD8^+^ T cell expansion through CD3/CD28 stimulation or DC co-culture. **b** A CD8^+^ T cell expansion assay of co-culturing naïve Pmel-1 CD8^+^ T cells with Ly5.1^+^ gp100-specified BMDCs. After 48-h co-culture, the 13% increase in number of generation (G) ≥ 2, CD25^+ ^CD90.1^+ ^CD8^+^ T cells in control and the 20.9% increase in mH-added group. **c** The cell number of CD25^+ ^CD8^+^ T cells in G ≥ 2 cell generation. **d** The CD8^+^ T cell expansion assay of stimulating naïve Pmel-1 CD8^+^ T cells by using anti-CD3 antibody and anti-CD28 antibody. After 72-h stimulation, the 55.4% increase in number of G ≥ 2, CD25^+ ^CD90.1^+ ^CD8^+^ T cells in control and the 53.4% increase in mH-added group. Each result is representative of three independent experiments. Data are presented as mean ± SEM. A Student’s *t*-test is used to compare the statistically significant difference between the mH-added and mH-unadded groups

To imitate the in vivo CD8^+^ T cell expansion, we first generated a CD8^+^ T cell expansion assay by co-culturing Pmel-1 CD8^+^ T cells with gp100-pulsed BMDCs in vitro (Fig. 4a). After 48-h co-culture, we observed a 13% of proliferating Pmel-1 CD8^+^ T cells (CD25^+^ CD90.1^+^ CD8^+^ T cells) in control group and a 20.9% of proliferating Pmel-1 CD8^+^ T cells in mH-added group (Fig. 4b). There was an increased number of proliferating Pmel-1 CD8^+^ T cells in mH-added group relative to unadded control (*p* = 0.003) (Fig. 4c), suggesting that HMGN1 might enhance the CD8^+^ T cell proliferation in the presence of DCs.

To investigate whether DCs are important to support HMGN1-dependent proliferation of CD8^+^ T cells, we next generated another CD8^+^ T cell expansion assay by stimulating Pmel-1 CD8^+^ T cells with anti-CD3/CD28 antibodies. After 72-h stimulation, we observed a 55.4% of proliferating Pmel-1 CD8^+^ T cells in control group and a 53.4% of proliferating Pmel-1 CD8^+^ T cells in mH-added group (Fig. 4d). There was no significant difference between control and mH-added groups. Collectively, these results suggest that HMGN1 promotes DC-dependent CD8^+^ T cell proliferation.

### HMGN1/αCD4 treatment synergistically reduced exhausted CD8^+^ T cells in tumor

Following antigen exposure, CD8^+^ T cells proliferate and differentiate into effector cells, then most of effector cells die off, leaving a small fraction of these cells to become memory cells. However, long-term antigen exposure converts CD8^+^ T cells into exhausted cells in tumor microenvironment. These exhausted CD8^+^ T cells express co-inhibitory molecules (e.g., PD-1, LAG-3, TIM-3, TIGIT) and lose the ability to produce multiple cytokines such as interferon (IFN)-γ, tumor necrosis factor (TNF)-α, and interleukin (IL)-2, namely they lose anti-tumor activities [32–35].

**Fig. 5:**
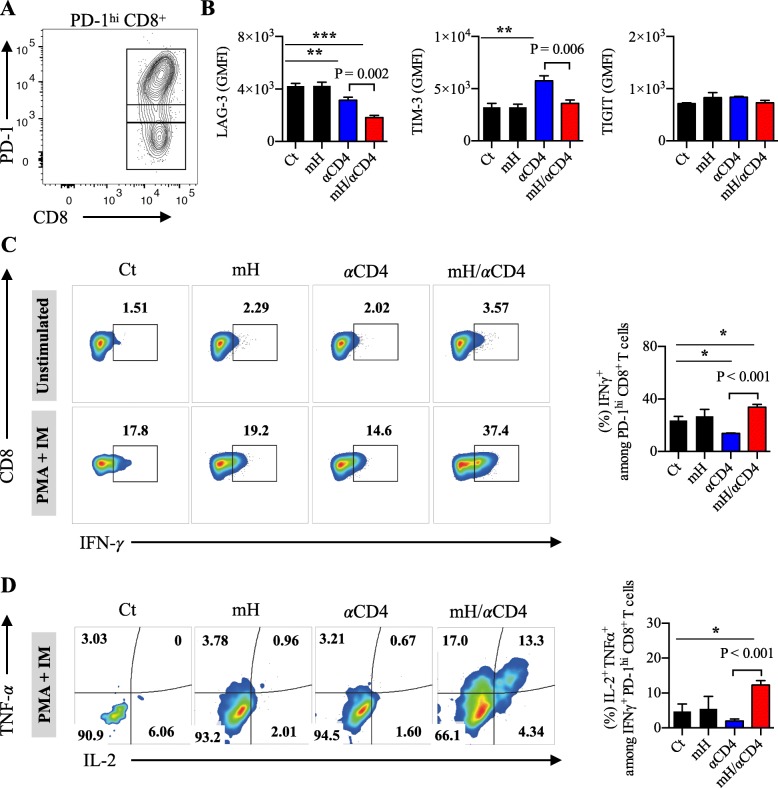
HMGN1/αCD4 treatment synergistically reduced exhausted CD8^+^ T cells in the tumor. **a** The compartment of PD-1^hi^, PD-1^lo^, PD-1^−^ CD8^+^ T cells in the tumor. **b** The expression level of LAG-3, TIM-3, and TIGIT on PD-1^hi^ CD8^+^ T cells. **c** The compartment and frequency of IFN-γ^+^ among in PD-1^+^ CD8^+^ T cells. **d** The compartment and frequency of IL-2^+^ TNF-α^+^ among in IFN-γ^+^ PD-1^+^ CD8^+^ T cells. Each result is representative of three independent experiments with at least four mice per group. Data are presented as mean ± SEM. *, *P* < 0.05, **, *P* < 0.01, ***, *P* < 0.001 for a dunnett’s post hoc test (compared with control); *p* values in the figure indicate Student’s *t*-test comparing mH/αCD4-treated and αCD4-treated groups. Geometric mean fluorescence intensity (GMFI)

To determine whether mH/αCD4 treatment is able to restore the effector functions of those exhausted CD8^+^ T cells, we analyzed the expression of co-inhibitory molecules and the ability of cytokine production from these PD-1^hi^ CD8^+^ tumor-infiltrating T cells on day17 after tumor inoculation (Fig. 5a). We observed that the expression of LAG-3 and TIM-3 on the PD-1^hi^ CD8^+^ T cells significantly decreased in the mH/αCD4-treated mice compared to the untreated control mice (*p* < 0.001) and the αCD4-treated mice (*p* = 0.002), whereas no significant difference was observed in the TIGIT expression among treatment groups (Fig. 5b). The number and percentage of LAG-3^+^ PD-1^+^ CD8^+^ T cells decreased in the mH/αCD4 treated mice relative to that of the αCD4 treated mice (*p* = 0.04) (S6A, B), whereas the percentage of TIM-3^+^ PD-1^+^ or TIGIT^+^ PD-1^+^ was equivalent among treatment groups (Additional file 1: Figure S6C). In addition, the proportion of the IFN-γ-, TNF-α-, and IL-2-producing multifunctional population within the PD-1^hi^ CD8^+^ T cells was higher in the mH/αCD4-treated mice than in the αCD4 treated (*P* < 0.001) or untreated control (*P* < 0.001) mice (Fig. 5c, d). Collectively, these results suggested that the HMGN1/αCD4 treatment reduces the level of co-inhibitory molecules in these exhausted CD8^+^ T cells, increases their effector functions, and decreases the proportion of exhausted CD8^+^ T cells in the tumor.

### Transcriptome analysis revealed the activation signatures of CD8^+^ T cells after HMGN1/αCD4 treatment

**Fig. 6:**
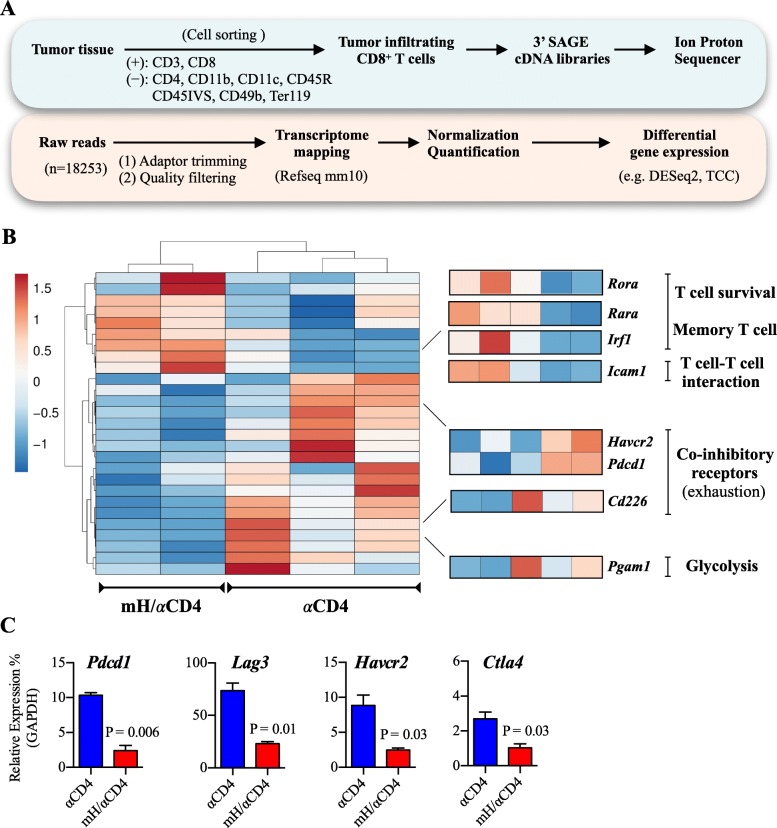
CD8^+^ T cell transcriptome analysis revealed the promotion of activation after HMGN1/αCD4 treatment. **a** Analysis scheme of transcriptome analysis of CD8^+^ T cells in tumor-bearing mice. **b** Heatmap representation of DEGs that associated with T cell function-related significantly-enriched GO groups genes. Each column represents group, whereas each row represents an individual gene. Z-score of module groups is shown on the left bottom of the heatmap. **c** qPCR analysis of expression changes of PD-1(*Pdcd1*), TIM-3 (*Havcr2*), LAG-3 (*Lag3*), and CTLA-4(*Ctla4*). The expression levels of all target mRNAs were normalized against the expression level of *Gapdh* in each sample. Data are presented as mean ± SEM. A Student’s *t*-test is used to compare the statistically significant difference between mH/αCD4-treated and αCD4-treated groups

To gain further insight into the phenotypic changes of CD8^+^ T cells during HMGN1/αCD4 treatment, we performed a transcriptome analysis on tumor-infiltrating CD8^+^ T cells on day 16. We identified 517 differentially expressed genes (DEGs) with adjusted *p* value less than 0.05 and a fold-change of ≥2 between at least two samples. In comparison with αCD4 treatment group, CD8^+^ T cells had 153 up-regulated and 364 down-regulated DEGs in mH/αCD4 treatment group (Fig. 6a). GO analysis revealed that these 153 up-regulated genes significantly included T cell activation-, chemokine production-, responses to interferons-, and ribosome related translation processes-related genes (Additional file 1: Figure S7). Particularly, these genes included cytotoxic function-related (*Gzmm*), chemotaxis-related (*Ccl5*), interferon-related (*Ifitm1*, *Ifitm2*, *Ifitm3*), T cell survival and memory-related (*Rora*, *Rara*, *Irf1*), and T cell-T cell interaction-related (*Icam1*) genes (Fig. 6b). In contrast, those 364 down-regulated genes significantly included cell cycle-, cell division-, telomere maintenance- and chromosome organization processes-related genes (Additional file 1:Figure S7). These genes included co-inhibitory-related (*Pdcd1*, *Havcr2, Cd226*), glycolysis-related (*Pgam1*), telomere-related (*Nek2*, *Hnrnpa1*, *Hnrnpc*, *Dkc1*, *Cct2*), and cell cycle/ cell division-related (*Atr*, *Bub1b*, *Ccnb2*, *Ccnd3*, *Ccng2*, *Cdc27*, *Cdkn1b*, *Chek2*, *Mcm6*, *Pcna*) genes (Fig. 6b).

Consistent with the transcriptome data and the flow cytometric data in Fig. 5, qPCR results also revealed down-regulation of co-inhibitory receptor-related genes (PD-1 (*Pdcd1*), TIM-3 (*Havcr2*), LAG-3 (*Lag3*), and CTLA-4 (*Ctla4*)) in CD8^+^ T cells after mH/αCD4 treatment relative to that of αCD4 treatment (Fig. 6c). Collectively, these results suggested that HMGN1 treatment might promote CD8^+^ T cell activation and effector functions, and sequentially suppressed the cell cycle and differentiation-related processes.

## Discussion

Comparing to intratumoral injection of HMGN1 (at 10 μg/ injection/ mice) in previous reports [10, 11], an optimal therapeutic dose (in the range: 0.08 to 0.4 μg/ injection) of HMGN1 was used for combination therapy in our study, which was beyond our expectations. Previously we demonstrated that the depletion of CD4^+^ T cells enhances CD8^+^ T cell proliferation in the draining lymph node of tumor-bearing mice [2], and that intratumoral injection of HMGN1 promotes the recruitment and maturation of DCs, thus resulting in the enhancement of endogenous immune responses [7]. In the present study, we observed that those mice receiving intraperitoneal administration of low-dose HMGN1 with αCD4 had an increasing number of tumor-infiltrating CD8^+^ T cells with the less exhausted phenotypes and the capacity to produce multiple cytokines. The cellular and molecular mechanisms underlying the increase in the number of functional CD8^+^ T cells after HMGN1/αCD4 combination treatment remain elusive. However, it is likely that the depletion of the Foxp3^+^ CD4^+^ regulatory T cells, which suppresses CD8^+^ T cell expansion in the draining lymph node through the cytotoxic T-lymphocyte-associated antigen-4 (CTLA-4) medicated downregulation of B7 co-stimulatory molecules in DCs, may contribute to the increased number of tumor-infiltrating CD8^+^ T cells.

In term of the function of endogenous HMGN1 within nucleus, Michael Bustin and his colleague reported that lack of endogenous HMGN1 in mouse embryonic fibroblasts increased the sensitivity to ionizing radiation, and enhanced the tumorigenesis including the proliferation, colony formation and tumor development. Lack of endogenous HMGN1 affects histone modifications which relate to double-strand breaks repair, thus resulting in the abnormal chromatin formation. The abnormal chromatin formation causes high radiosensitivity and tumorigenesis in *Hmgn1*^*−/−*^ cells [36]. Moreover, *Hmgn1*^*−/−*^ mice without endogenous HMGN1 displayed lower number of CD8^+^ T cells and higher tumor growth rate relative to *Hmgn1*^+/+^ mice [7]. All these findings indicate that lack of endogenous HMGN1 is likely to contribute to tumor progression.

On the other hand, there has been an increasing interest in the interplay between physiological function of extracellular HMGN1 and immune activation of cells. In 2012, we reported that extracellular HMGN1 binds to toll-like receptor (TLR)-4 on DCs to induce pro-inflammatory immune responses. Once TLR-4 downstream signaling pathway was blocked, HMGN1 lost most of the ability to promote DC activation and inflammation, suggesting that TLR-4 is a receptor for extracellular HMGN1 [6]. Furthermore, another HMG family protein HMGB1 is reported to induce chemotaxis and inflammation through the receptor for advanced glycation end products (RAGE) or TLR-2 [37–40] . The interaction of HMGB1 and RAGE triggers the activation of mitogen-activated protein kinase (MAPK) pathway, which activates downstream signaling molecule nuclear factor (NF)-*κ*B to induce inflammation [41]. It is likely that TLR2/4 and RAGE would serve as HMGN1 receptor, though the effective doses of HMGN1 we used in this study were extremely lower than the doses described in previous reports, which suggesting the existence of additional specific receptor for HMGN1.

In the context of CD8^+^ effector and memory T-cell differentiation, our transcriptomic analysis revealed that CD8^+^ T cells expressed more cytotoxic and chemotactic molecules with their active phenotype after HMGN1/αCD4 treatment than αCD4 treatment; however, those CD8^+^ T cells also had slow-dividing cell feature with low expression of cell cycle/cell division-associated genes. In other words, those CD8^+^ T cells were undergoing a cell intrinsic process to promote TCR stimulation, co-stimulatory signals, and cytokine releasing by downregulating cell cycle phase and/or metabolic activity, which resembles memory T cells [42, 43]. This result is consistent with our finding that the number of CD44^hi^ PD-1^+^ CD8^+^ T cells increased in the tumor. Those CD44^hi^ PD-1^+^ CD8^+^ T cells with memory pattern have high sensitivity to antigen stimulation, which resulting in a rapid proliferation and a high-level cytotoxic and cytokine productions [44–46]. Thus, the enhancement of CD8^+^ T cell signatures possibly explains the reason why those mice receiving HMGN1/αCD4 treatment would be able to resist tumor progression and recurrence.

In addition to the combination therapy of HMGN1 with αCD4, there is another combination therapy related to HMGN1. The TheraVac is composed of HMGN1, R-848 (also known as Resiquimod) and immune checkpoint blockades (e.g. anti-PD-L1 antibody, anti-CTLA4 antibody). In a hepatocellular tumor model (Hepa1–6), mice treated with TheraVac had the increased number of cytotoxic T lymphocytes in the draining lymph node and tumor [10, 11]. However, the number of CD4^+^ T cells also increased in tumor after HMGN1/R848 or TheraVac treatment, which raises a possibility to cause immunosuppression by CD4^+^ T regulatory cells. Thus, combination therapy of TheraVac with αCD4 might be an effective method to limit the function and number of CD4^+^ immunosuppressive cells in short-term. Now, we are in the process of conducting phase I clinical trial of a humanized anti-human CD4 depleting antibody IT1208 in Japan to promote the anti-tumor effect in human. In preclinical studies in nonhuman primates, no serious adverse effects were detected after several weeks of treatment with our humanized anti human CD4 depleting antibody.

## Conclusion

Our study represents a novel combination therapy of low-dose HMGN1 with αCD4 to exert robust anti-tumor effects in mice. The combination therapy brings synergistic effect of enhancing tumor-associated CD8^+^ T cell expansion together with reduced exhaustion phenotype, with resultant robust anti-tumor effects. Combination therapy of HMGN1/αCD4 might be a promising strategy to treat patients with solid tumors.

## Additional file

Additional file 1: **Figure S1.** The purification of recombinant HMGN1 proteins. **Figure S2.** Administration of human HMGN1 (hH) with anti-CD4 depleting antibody (αCD4) exerted anti-tumor effects in mice. **Figure S3.** Administration of murine HMGN1 with anti-CD4 antibody exerted anti-tumor effects in melanoma model. (A). **Figure S4.** Flow cytometry analysis of T cell populations in the draining lymph node (dLN) from Colon26 tumor-bearing mice on day 13 after tumor inoculation. **Figure S5.** Gating scheme of CCR7^+^ CD11b^+^ migratory dendritic cells (DCs). **Figure S6.** Flow cytometry analysis of exhausted CD8^+^ T cell populations in the tumor from tumor-bearing mice on day 17 after treatment. **Figure S7.** CD8 T cell transcriptome analysis revealed the promotion of activation after HMGN1/αCD4 treatment. **Method S1.** Production and purification of HMGN1 in *E. coli*. **Method S2.** 3’end Serial Analysis of Gene Expression (SAGE) sequencing library preparation. **Table S1.** The list of recombinant proteins and peptides. **Table S2.** Antibodies for flow cytometry. **Table S3.** Primers for quantitative real-time PCR. **Table S4.** Gene ontology (GO) analysis of GO: biological processes and KEGG pathway. (PDF 2580 kb )

## Abbreviations

CCR: C-C chemokine receptor
CFSE: Carboxyfluorescein diacetate succinimidyl ester
CTLA-4: Cytotoxic T-lymphocyte-associated antigen-4
DAMP: Damage-associated molecular pattern
DC: Dendritic cell
DEG: Differentially expressed genes
FITC: Fluorescein isothiocyanate
GO: Gene Ontology
HMGN1: High mobility group nucleosome binding protein 1
IFN: Interferon
IL: Interleukin
KEGG: Kyoto Encyclopedia of Genes and Genomes
MAPK: Mitogen-activated protein kinase
NF: Nuclear factor
PD-1: Programmed cell death 1
PD-L1: Programmed cell death ligand 1
RAGE: Receptor for advanced glycation end product
TLR: Toll-like receptor
TNF: Tumor necrosis factor
αCD4: Anti-CD4 depleting antibody

## References

[1] Whiteside TL (2012). Disarming suppressor cells to improve immunotherapy. Cancer Immunol Immunother.

[2] Ueha S, Yokochi S, Ishiwata Y, Ogiwara H, Chand K, Nakajima T, Hachiga K, Shichino S, Terashima Y, Toda E (2015). Robust antitumor effects of combined anti-CD4-depleting antibody and anti-PD-1/PD-L1 immune checkpoint antibody treatment in mice. Cancer Immunol Res.

[3] Liu C, Workman CJ, Vignali DA (2016). Targeting regulatory T cells in tumors. FEBS J.

[4] Bianchi ME, Agresti A (2005). HMG proteins: dynamic players in gene regulation and differentiation. Curr Opin Genet Dev.

[5] Bianchi ME (2007). DAMPs, PAMPs and alarmins: all we need to know about danger. J Leukoc Biol.

[6] Yang D, Postnikov YV, Li Y, Tewary P, de la Rosa G, Wei F, Klinman D, Gioannini T, Weiss JP, Furusawa T (2012). High-mobility group nucleosome-binding protein 1 acts as an alarmin and is critical for lipopolysaccharide-induced immune responses. J Exp Med.

[7] Wei F, Yang D, Tewary P, Li Y, Li S, Chen X, Howard OM, Bustin M, Oppenheim JJ (2014). The Alarmin HMGN1 contributes to antitumor immunity and is a potent immunoadjuvant. Cancer Res.

[8] Yang D, Bustin M, Oppenheim JJ (2015). Harnessing the alarmin HMGN1 for anticancer therapy. Immunotherapy.

[9] Nie Y, Yang D, Oppenheim JJ (2016). Alarmins and antitumor immunity. Clin Ther.

[10] Han Z, Yang, Trivett A, Oppenheim JJ (2017). Therapeutic vaccine to cure large mouse hepatocellular carcinomas. Oncotarget.

[11] Nie Y, Yang, Trivett A, Han Z, Xin H, Chen X, Oppenheim JJ (2017). Development of a curative therapeutic vaccine (TheraVac) for the treatment of large established tumors. Sci Rep.

[12] Anderson KG, Mayer-Barber K, Sung H, Beura L, James BR, Taylor JJ, Qunaj L, Griffith TS, Vezys V, Barber DL (2014). Intravascular staining for discrimination of vascular and tissue leukocytes. Nat Protoc.

[13] Sawanobori Y, Ueha S, Kurachi M, Shimaoka T, Talmadge JE, Abe J, Shono Y, Kitabatake M, Kakimi K, Mukaida N (2008). Chemokine-mediated rapid turnover of myeloid-derived suppressor cells in tumor-bearing mice. Blood.

[14] Shand FH, Ueha S, Otsuji M, Koid SS, Shichino S, Tsukui T, Kosugi-Kanaya M, Abe J, Tomura M, Ziogas J (2014). Tracking of intertissue migration reveals the origins of tumor-infiltrating monocytes. Proc Natl Acad Sci U S A.

[15] Bolger AM, Lohse M, Usadel B (2014). Trimmomatic: a flexible trimmer for Illumina sequence data. Bioinformatics.

[16] Schmieder R, Edwards R (2011). Quality control and preprocessing of metagenomic datasets. Bioinformatics.

[17] Sun J, Nishiyama T, Shimizu K, Kadota K (2013). TCC: an R package for comparing tag count data with robust normalization strategies. BMC Bioinformatics.

[18] Love MI, Huber W, Anders S (2014). Moderated estimation of fold change and dispersion for RNA-seq data with DESeq2. Genome Biol.

[19] Robinson MD, McCarthy DJ, Smyth GK (2010). edgeR: a Bioconductor package for differential expression analysis of digital gene expression data. Bioinformatics.

[20] Bindea G, Mlecnik B, Hackl H, Charoentong P, Tosolini M, Kirilovsky A, Fridman WH, Pages F, Trajanoski Z, Galon J (2009). ClueGO: a Cytoscape plug-in to decipher functionally grouped gene ontology and pathway annotation networks. Bioinformatics.

[21] Saito R, Smoot ME, Ono K, Ruscheinski J, Wang PL, Lotia S, Pico AR, Bader GD, Ideker T (2012). A travel guide to Cytoscape plugins. Nat Methods.

[22] Mi H, Muruganujan A, Casagrande JT, Thomas PD (2013). Large-scale gene function analysis with the PANTHER classification system. Nat Protoc.

[23] Kanehisa M, Furumichi M, Tanabe M, Sato Y, Morishima K (2017). KEGG: new perspectives on genomes, pathways, diseases and drugs. Nucleic Acids Res.

[24] Gros A, Robbins PF, Yao X, Li YF, Turcotte S, Tran E, Wunderlich JR, Mixon A, Farid S, Dudley ME (2014). PD-1 identifies the patient-specific CD8(+) tumor-reactive repertoire infiltrating human tumors. J Clin Invest.

[25] Inozume T, Hanada K, Wang QJ, Ahmadzadeh M, Wunderlich JR, Rosenberg SA, Yang JC (2010). Selection of CD8+PD-1+ lymphocytes in fresh human melanomas enriches for tumor-reactive T cells. J Immunother.

[26] Ye Q, Song DG, Poussin M, Yamamoto T, Best A, Li C, Coukos G, Powell DJ (2014). CD137 accurately identifies and enriches for naturally occurring tumor-reactive T cells in tumor. Clin Cancer Res.

[27] Bellavance EC, Kohlhapp FJ, Zloza A, O'Sullivan JA, McCracken J, Jagoda MC, Lacek AT, Posner MC, Guevara-Patino JA (2011). Development of tumor-infiltrating CD8+ T cell memory precursor effector cells and antimelanoma memory responses are the result of vaccination and TGF-beta blockade during the perioperative period of tumor resection. J Immunol.

[28] Stout RD, Suttles J (1992). T cells bearing the CD44hi "memory" phenotype display characteristics of activated cells in G1 stage of cell cycle. Cell Immunol.

[29] Hirao M, Onai N, Hiroishi K, Watkins SC, Matsushima K, Robbins PD, Lotze MT, Tahara H (2000). CC chemokine receptor-7 on dendritic cells is induced after interaction with apoptotic tumor cells: critical role in migration from the tumor site to draining lymph nodes. Cancer Res.

[30] Ohl L, Mohaupt M, Czeloth N, Hintzen G, Kiafard Z, Zwirner J, Blankenstein T, Henning G, Forster R (2004). CCR7 governs skin dendritic cell migration under inflammatory and steady-state conditions. Immunity.

[31] Roberts EW, Broz ML, Binnewies M, Headley MB, Nelson AE, Wolf DM, Kaisho T, Bogunovic D, Bhardwaj N, Krummel MF (2016). Critical role for CD103(+)/CD141(+) dendritic cells bearing CCR7 for tumor antigen trafficking and priming of T cell immunity in melanoma. Cancer Cell.

[32] Baumeister SH, Freeman GJ, Dranoff G, Sharpe AH (2016). Coinhibitory pathways in immunotherapy for Cancer. Annu Rev Immunol.

[33] Anderson AC, Joller N, Kuchroo VK (2016). Lag-3, Tim-3, and TIGIT: co-inhibitory receptors with specialized functions in immune regulation. Immunity.

[34] Wherry EJ (2011). T cell exhaustion. Nat Immunol.

[35] Hashimoto M, Kamphorst AO, Im SJ, Kissick HT, Pillai RN, Ramalingam SS, Araki K, Ahmed R (2018). CD8 T cell exhaustion in chronic infection and Cancer: opportunities for interventions. Annu Rev Med.

[36] Birger Y, Catez F, Furusawa T, Lim JH, Prymakowska-Bosak M, West KL, Postnikov YV, Haines DC, Bustin M (2005). Increased tumorigenicity and sensitivity to ionizing radiation upon loss of chromosomal protein HMGN1. Cancer Res.

[37] Degryse B, Bonaldi T, Scaffidi P, Muller S, Resnati M, Sanvito F, Arrigoni G, Bianchi ME (2001). The high mobility group (HMG) boxes of the nuclear protein HMG1 induce chemotaxis and cytoskeleton reorganization in rat smooth muscle cells. J Cell Biol.

[38] Yu M, Wang H, Ding A, Golenbock DT, Latz E, Czura CJ, Fenton MJ, Tracey KJ, Yang H (2006). HMGB1 signals through toll-like receptor (TLR) 4 and TLR2. Shock.

[39] Yang H, Wang H, Czura CJ, Tracey KJ (2005). The cytokine activity of HMGB1. J Leukoc Biol.

[40] Hori O, Brett J, Slattery T, Cao R, Zhang J, Chen JX, Nagashima M, Lundh ER, Vijay S, Nitecki D (1995). The receptor for advanced glycation end products (RAGE) is a cellular binding site for amphoterin. Mediation of neurite outgrowth and co-expression of rage and amphoterin in the developing nervous system. J Biol Chem.

[41] Park JS, Arcaroli J, Yum HK, Yang H, Wang H, Yang KY, Choe KH, Strassheim D, Pitts TM, Tracey KJ (2003). Activation of gene expression in human neutrophils by high mobility group box 1 protein. Am J Physiol Cell Physiol.

[42] Kinjyo I, Qin J, Tan SY, Wellard CJ, Mrass P, Ritchie W, Doi A, Cavanagh LL, Tomura M, Sakaue-Sawano A (2015). Real-time tracking of cell cycle progression during CD8+ effector and memory T-cell differentiation. Nat Commun.

[43] Marchingo JM, Kan A, Sutherland RM, Duffy KR, Wellard CJ, Belz GT, Lew AM, Dowling MR, Heinzel S, Hodgkin PD (2014). T cell signaling. Antigen affinity, costimulation, and cytokine inputs sum linearly to amplify T cell expansion. Science.

[44] Haluszczak C, Akue AD, Hamilton SE, Johnson LD, Pujanauski L, Teodorovic L, Jameson SC, Kedl RM (2009). The antigen-specific CD8+ T cell repertoire in unimmunized mice includes memory phenotype cells bearing markers of homeostatic expansion. J Exp Med.

[45] Seder RA, Ahmed R (2003). Similarities and differences in CD4+ and CD8+ effector and memory T cell generation. Nat Immunol.

[46] Kalia V, Sarkar S, Gourley TS, Rouse BT, Ahmed R (2006). Differentiation of memory B and T cells. Curr Opin Immunol.

