# Supplementary material for: Combined treatment with HMGN1 and anti-CD4 depleting antibody reverses T cell exhaustion and exerts robust anti-tumor effects in mice
Source: J Immunother Cancer. 2019 Jan 29;7:21. doi: 10.1186/s40425-019-0503-6 (PMC6352494; doi:10.1186/s40425-019-0503-6)
Supplement: Supplementary file 1 — Figure S1. The purification of recombinant HMGN1 proteins. Figure S2. Administration of human HMGN1 (hH) with anti-CD4 depleting antibody (αCD4) exerted anti-tumor effects in mice. Figure S3. Administration of murine HMGN1 with anti-CD4 antibody exerted anti-tumor effects in melanoma model. (A). Figure S4. Flow cytometry analysis of T cell populations in the draining lymph node (dLN) from Colon26 tumor-bearing mice on day 13 after tumor inoculation. Figure S5. Gating scheme of CCR7+ CD11b+ migratory dendritic cells (DCs). Figure S6. Flow cytometry analysis of exhausted CD8+ T cell populations in the tumor from tumor-bearing mice on day 17 after treatment. Figure S7. CD8 T cell transcriptome analysis revealed the promotion of activation after HMGN1/αCD4 treatment. Method S1. Production and purification of HMGN1 in E. coli. Method S2. 3’end Serial Analysis of Gene Expression (SAGE) sequencing library preparation. Table S1. The list of recombinant proteins and peptides. Table S2. Antibodies for flow cytometry. Table S3. Primers for quantitative real-time PCR. Table S4. Gene ontology (GO) analysis of GO: biological processes and KEGG pathway. (PDF 2580 kb ) [file 40425_2019_503_MOESM1_ESM.pdf]

Supplementary materials

Combined treatment with HMGN1 and anti-CD4 depleting antibody reverses T cell exhaustion and exerts robust anti-tumor effects in mice

Chang-Yu Chen, Satoshi Ueha, Yoshiro Ishiwata, Shoji Yokochi, De Yang, Joost J. Oppenheim, Haru Ogiwara, Shigeyuki Shichino, Shungo Deshimaru, Francis H.W. Shand, Shiro Shibayama, Kouji Matsushima

7 figures, 3 methods, and 4 tables

Supplementary figures

Figure S1

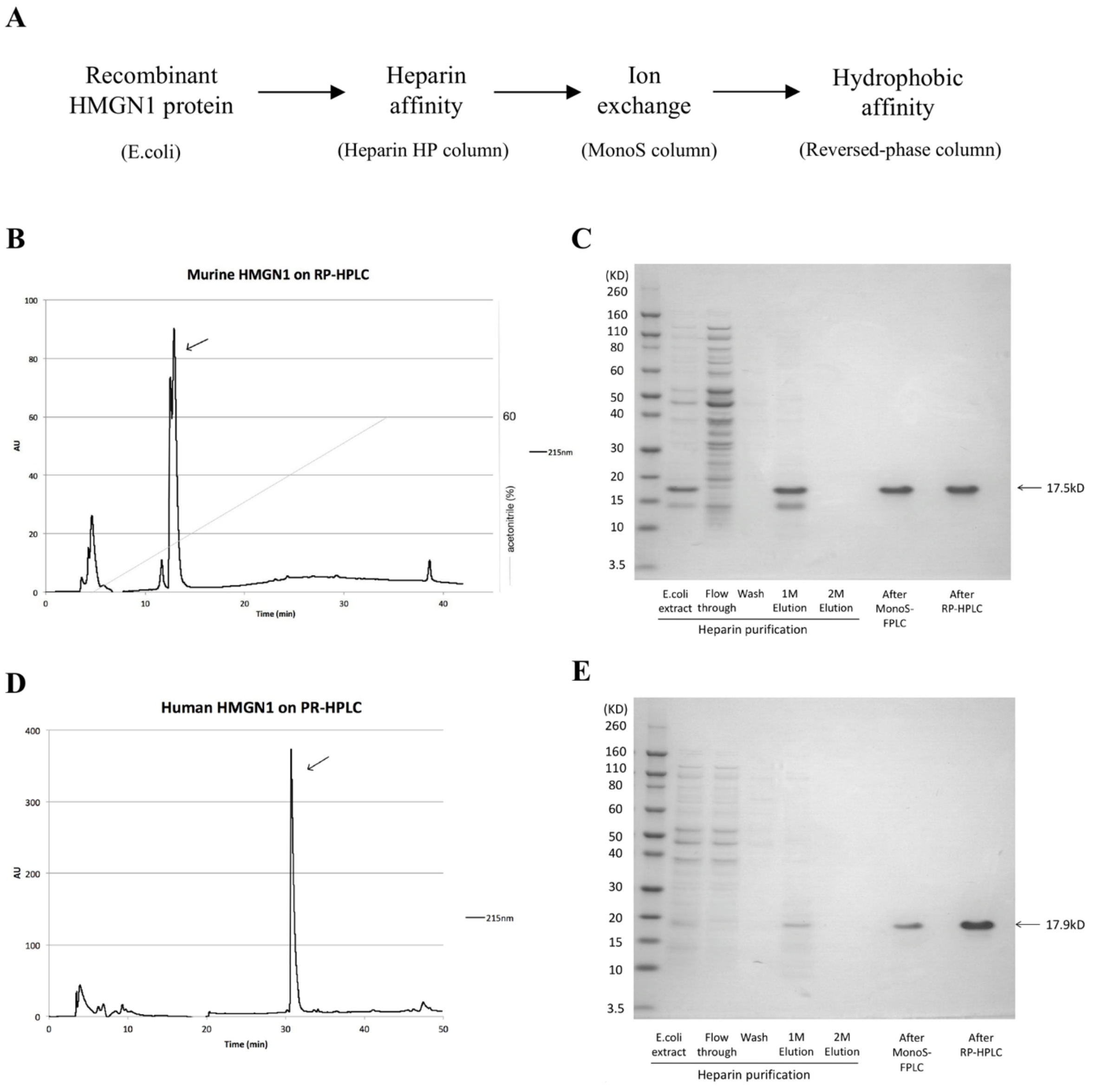

Figure S1. The purification of recombinant HMGN1 proteins. (A) The flowchart of purifying recombinant HMGN1 protein. **(B, D)** In reverse phase HPLC (RP-HPLC), the linear fractions with the highest absorbance unit (AU) were collected, and identified as murine HMGN1 (mH) or human HMGN1 (hH) by N-terminal amino acid sequencing. **(C, E)** MES buffered 4-12% Bis-Tris SDS-PAGE analysis of stepwise purification of mH and hH.

Figure S2

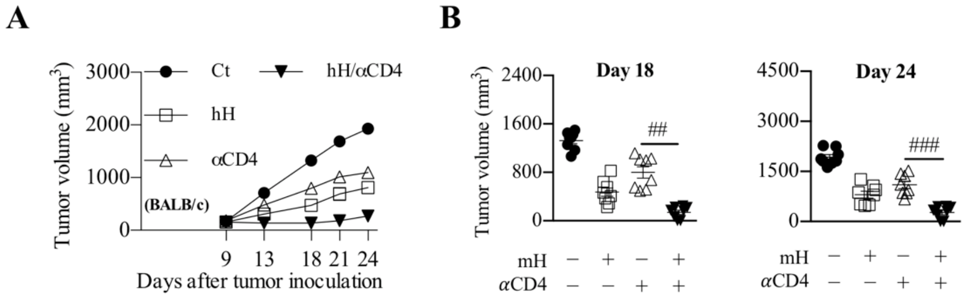

Figure S2. Administration of human HMGN1 (hH) with anti-CD4 depleting antibody (αCD4) exerted anti-tumor effects in mice. The hH (at a dose of 0.16 μg/ injection) was administered intraperitoneally on days 9, 14, 17, and 20 after tumor inoculation. The αCD4 (at a dose of 200 μg/ injection) was injected intraperitoneally on days 5 and 9 after tumor inoculation. **(A)** The quantitative analysis of tumor growth and tumor progression for each mouse until 24 days after treatment. **(B)** The tumor volume of each mouse on day 18 and day 24. Tumor growth is representative of three independent experiments with at least eight mice per group. Data are presented as mean ± SEM. #, $P<0.05$, ##, $P<0.01$, ###, $p<0.001$ for a Student's *t*-test comparing mH/αCD4-treated and αCD4-treated groups.

Figure S3

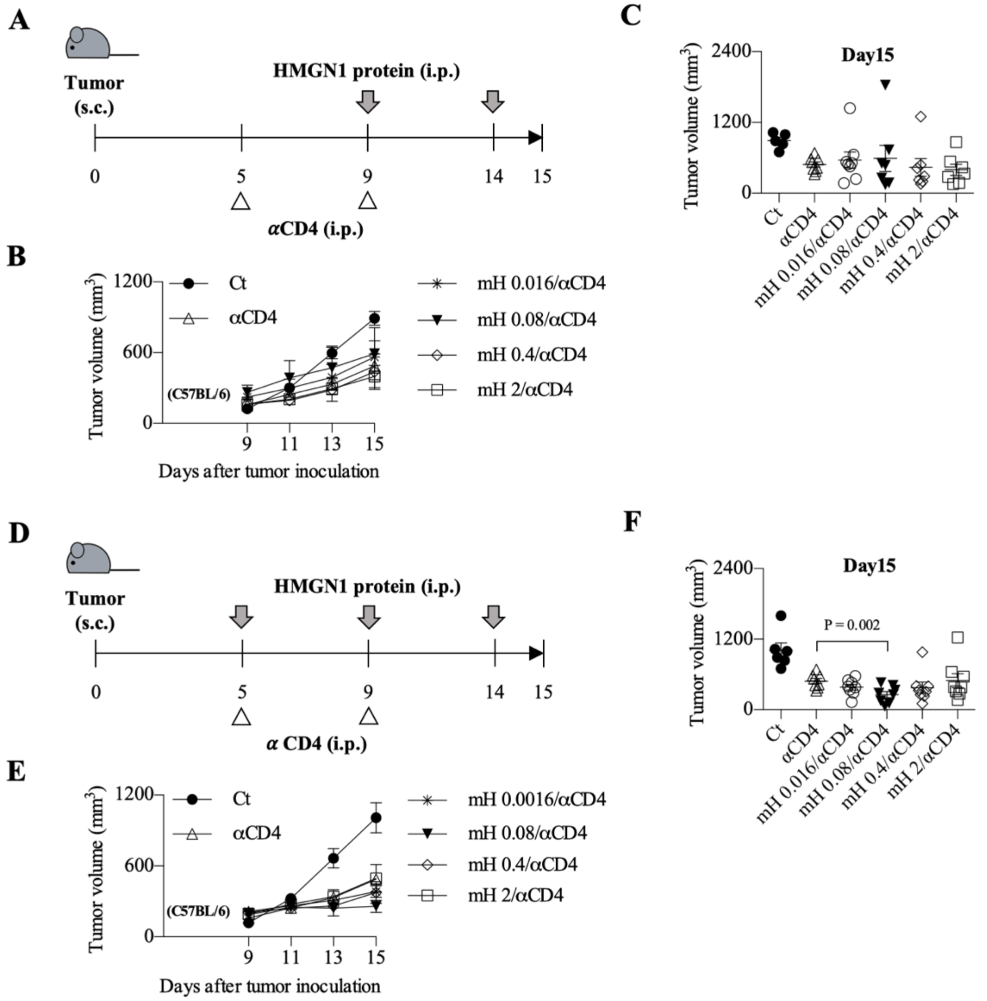

Figure S3. Administration of murine HMGN1 with anti-CD4 antibody exerted anti-tumor effects in melanoma model. (A) The administration of twice αCD4 on day 5, 9 and twice mHMGN1 on day 9, 14. **(B, C)** The tumor growth rate and the tumor volume after mHMGN1/αCD4 treatment. **(D)** The optimized administration of twice αCD4 on days 5, 9 and three times mHMGN1 on days 5, 9, 14. **(E, F)** The tumor growth rate and the tumor volume after optimized mHMGN1/αCD4 treatment. Tumor growth is representative of three independent experiments with at least eight mice per group. Data are presented as mean ± SEM.

Figure S4

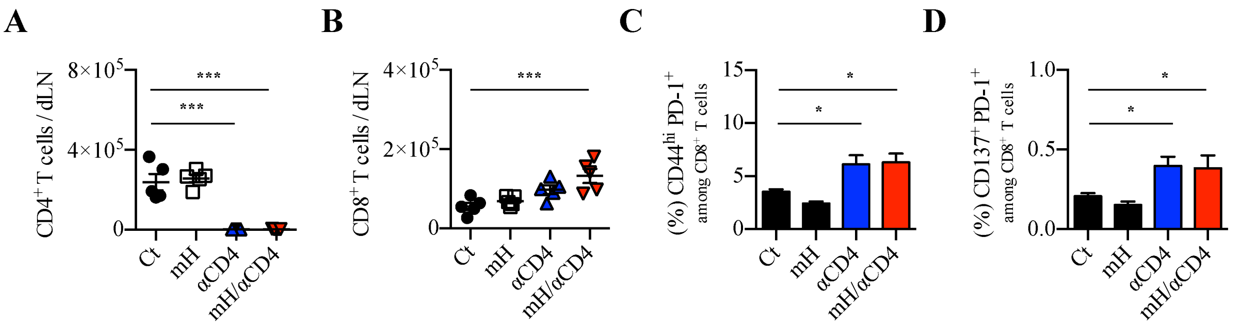

Figure S4. Flow cytometry analysis of T cell populations in the draining lymph node (dLN) from Colon26 tumor-bearing mice on day 13 after tumor inoculation. (A) The number of $CD4^+$ T cells. **(B)** The number of $CD8^+$ T cells. (**C, D**) The percentile of $CD137^+$ PD-1^+ and $CD44^{hi}$ PD-1^+ among $CD8^+$ T cells. Each result is representative of three independent experiments with at least four mice per group. Data are presented as mean ± SEM. *, $P<0.05$, **, $P<0.01$, ***, $P<0.001$ for a dunnett's *post hoc* test (compared with control).

Figure S5

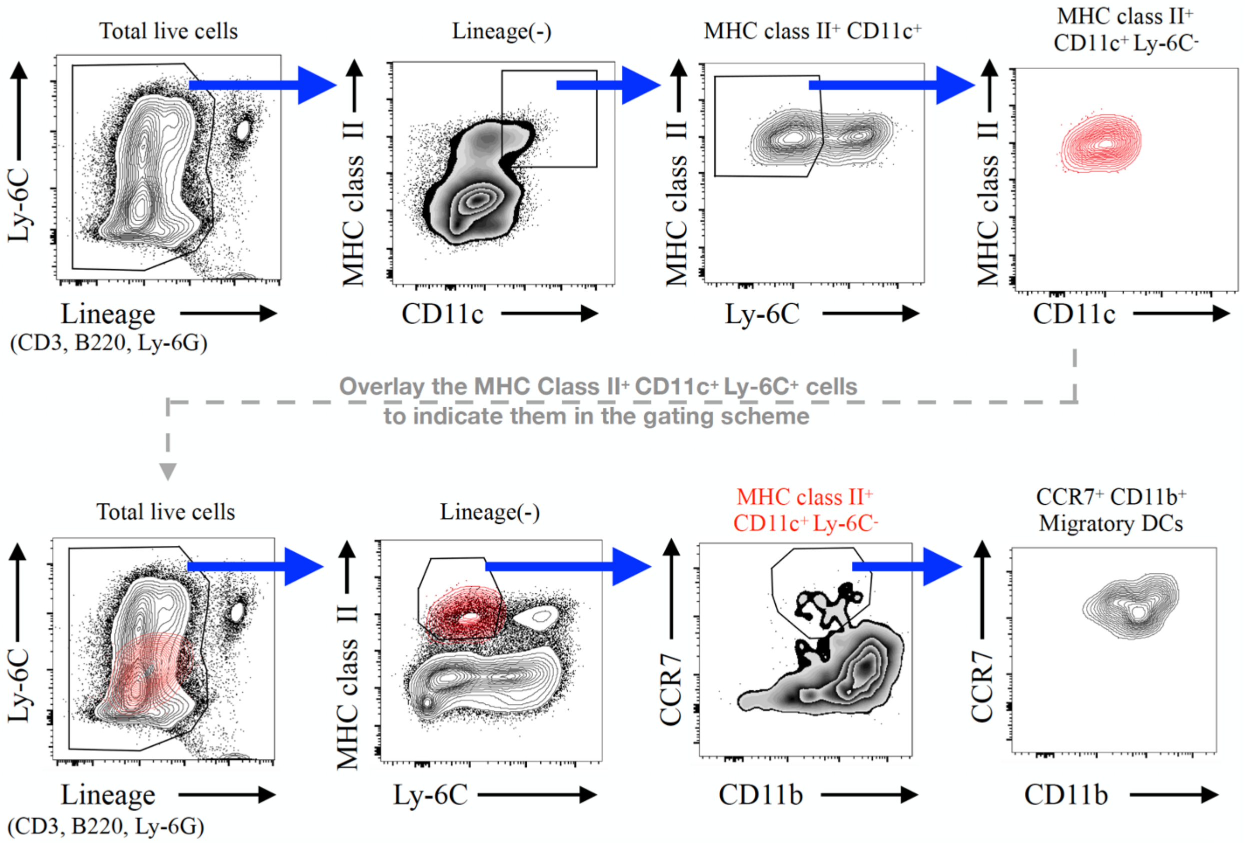

Figure S5. Gating scheme of CCR7^{+} CD11b^{+} migratory dendritic cells (DCs). Representative flow cytometry plots showing gating scheme for CCR7^{+} CD11b^{+} migratory DCs.

Figure S6

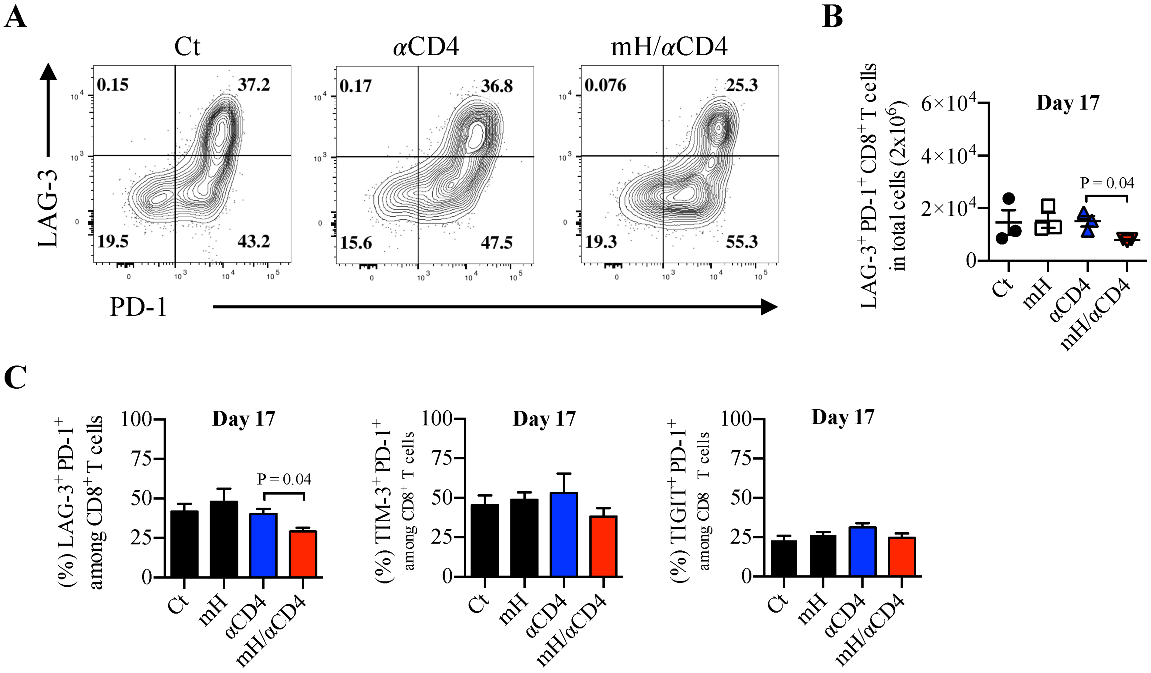

Figure S6. Flow cytometry analysis of exhausted $CD8^+$ T cell populations in the tumor from tumor-bearing mice on day 17 after treatment. (A, B) The compartment and the cell number of $LAG\text{-}3^+$ $PD\text{-}1^+$ $CD8^+$ T cells in the tumor. **(C)** The percentage of LAG^+ $PD\text{-}1^+$, $TIM\text{-}3^+$ $PD\text{-}1^+$, and $TIGIT^+$ $PD\text{-}1^+$ among $CD8^+$ T cells. Each result is representative of three independent experiments with at least four mice per group. Data are presented as mean ± SEM. p values in the figure indicate the Student's *t*-test comparing mH/αCD4-treated and αCD4-treated groups.

Figure S7

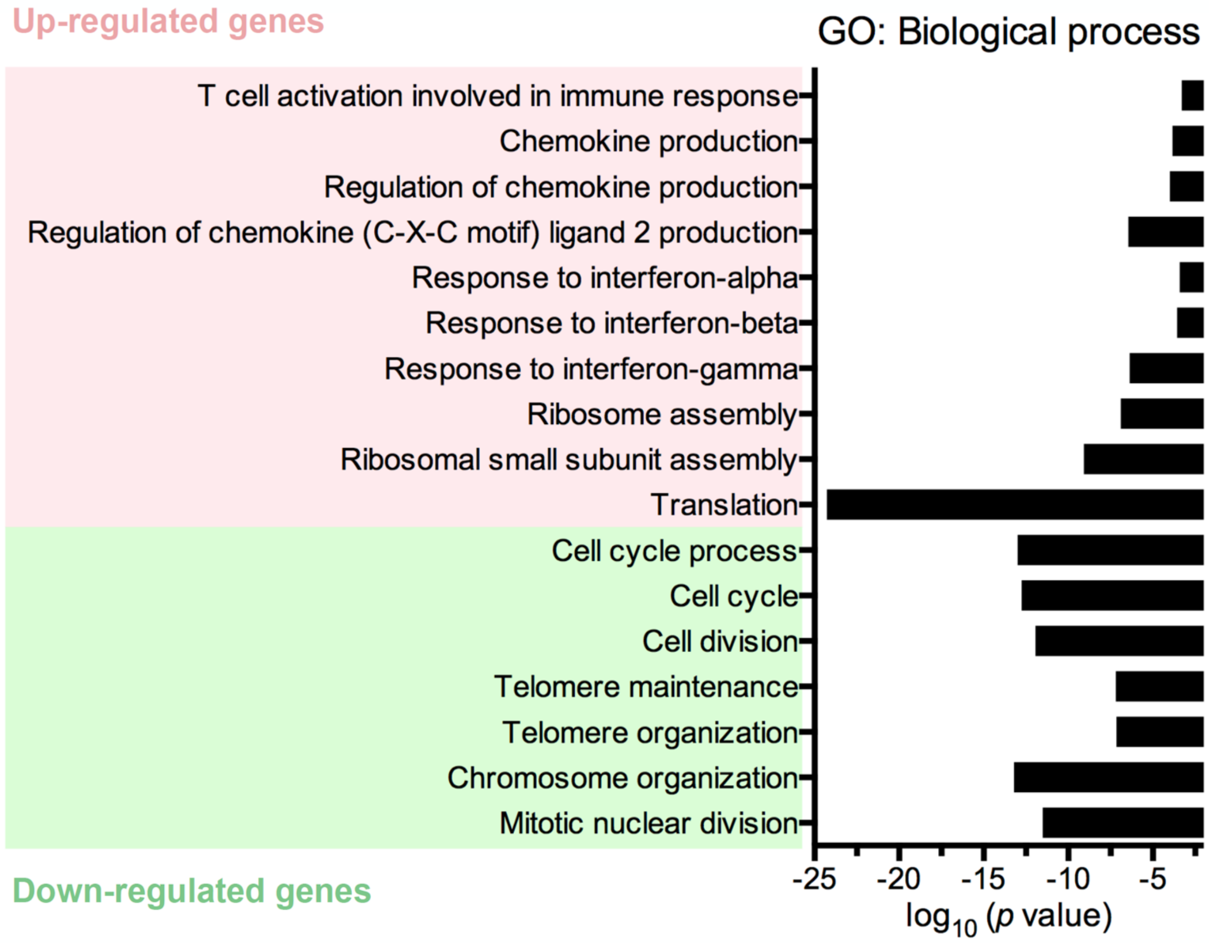

Figure S7. CD8 T cell transcriptome analysis revealed the promotion of activation after HMGN1/αCD4 treatment. Biological events associated with 517 DEGs (157 up-regulated and 364 down-regulated) were explored and grouped using Cytoscape and ClueGO plugin. Significantly enriched Gene ontology (GO) leading terms are shown on the left side of the graph. The statistical significance of each enrichment (*p* value, $\log_{10}$) was represented by bar graph.

Supplementary methods

Supplementary method 1. Production and purification of HMGN1 in E. coli

The expression pET-17b plasmid with murine HMGN1 coding region (122-412) or human HMGN1 coding region (205-507) was transformed into BL21 Star™ (DE3) E. coli (Thermo Fisher Scientific) by heat shock method. The single colony was picked up and pre-cultivated in a LB/ampicillin medium until the OD600 absorbance reaches 0.6. Added isopropyl β-D-1-thiogalactopyranoside (IPTG, final concentration equal to 1 mM) into culture medium and cultivated the cells for 4 hours. The cell pellet was collected by centrifugation at 15,000 x g for 5 min, and then the soluble proteins were extracted from cell pellet by sonication. To purify the recombinant HMGN1 protein from soluble proteins, the following procedures were administered to each subject in the order mentioned. (1) For heparin-affinity chromatography, the soluble proteins were applied to a HiTrap™ Heparin HP column (5 ml, GE Healthcare) equilibrated with 10 mM sodium phosphate buffer, pH 7.0 (NaPB) containing 0.2 M NaCl. After washing the column, HMGN1 was eluted by NaPB with 1 M NaCl. (2) For cation exchange chromatography, the eluted HMGN1 from heparin column was loaded into a Mono S HR 5/5 (5 x 50 mm, Pharmacia Biotech) equipped to a FPLC system (Pharmacia Biotech), equilibrated with NaPB buffer, and adjusted the flow rate to 1 ml/min. The HMGN1 fractions were eluted by a linear gradient from 0.2 to 1.0 M NaCl in NaPB. (3) For hydrophobic interaction chromatography, the HMGN1 fractions from MonoS-FPLC were directly referred to a reverse phase RP-304 column (4.6 x 205 mm, C4, BioRad) equipped to a HPLC system (Gilson). The HMGN1 was purified by a linear gradient of acetonitrile from 0 to 60% in water with 0.01% trifluoroacetic acid (TFA). The acetonitrile was further removed from the purified sample by freeze-drying. Highly purified recombinant HMGN1 was examined the endotoxin level and was kept frozen at -80°C.

Supplementary method 2. 3'end Serial Analysis of Gene Expression (SAGE) sequencing library preparation

Three minutes before collecting tumor tissues, intravascular leukocytes were stained by intravenous injection of FITC anti-CD45 antibody. $CD8^+$ T cells from tumor tissue were labeled with APC anti-CD8 antibody (clone: 53-6.7; BioLegend, USA), anti-APC microbeads (Miltenyl Biotec, Germany), and lineage antibody cocktail (FITC anti-CD11b, FITC anti-B220, FITC anti-CD49b, FITC anti-Ter119, and Pacific blue anti-CD4 antibodies). Labeled $CD8^+$ T cells were separated by using an autoMACS cell separator (Miltenyl Biotec, Germany). Then, $CD8^+$ T cells were further purified from lineage sorting ($CD45^-CD11b^-B220^-CD49b^-Ter119^-CD4^-CD8^+$) by FACSAria II Cell Sorter (BD Biosciences, USA).

Purity of isolated $CD8^+$ T cells among live cells was over 99% for RNA transcriptome analysis. Total RNA was extracted from 10,000 $CD8^+$ T cells of each sample and stored in lysis/binding buffer (Thermo Fisher Scientific, USA). Messenger RNA (mRNA) was isolated from total RNA by Dynabeads M-270 streptavidin with biotin-oligo $(dT)_{25}$ (Thermo Fisher Scientific, USA). First- and second-strand cDNA synthesis was performed by Superscript reverse transcriptase IV (Thermo Fisher Scientific, USA) and Kapa HiFi DNA polymerase (Roche, Switzerland). While capturing on the beads, cDNA was digested with restriction endonuclease NlaIII (Thermo Fisher Scientific, USA), which cleaved the 3'end CATG/GTAC restriction site. Each cDNA fragment carrying the 3'CATG overhang was ligated to an adapter carrying common sequence 1 (CS1) and EcoP15I recognition site (5'-CAGCCAG-3'), and sequentially digested by EcoP15I restriction endonuclease to generate a 27-bp sequence with 5' end overhang (2-bp). Next, another adaptor carrying CS2 and sequencing primer site was ligated to the 5' end overhang of 27-bp sequence, and the 46-bp ligation product was obtained by MinElute PCR purification kit (Qiagen, Germany). Finally, an 8-cycle PCR step was performed to enrich for the desired cDNA library molecules, and those cDNA library products were purified by size selection using AMPure XP beads (Beckman Coulter, USA). We used Ion Torrent (Thermo Fisher Scientific, USA) to prepare cDNA template, the template which further analyzed by Ion Proton Sequencer (Thermo Fisher Scientific, USA).

Supplementary tables

Table S1 │The list of recombinant proteins and peptides

Species	Description	Abbreviation	Amino acid sequence
Murine	HMGN1 (1-96 a.a)	mH	1- MPKRKVSADGAAKAEPKRRS<br>21- ARLSAKPAPAKVDAKPKKAA<br>41- GKDKASDKKVQIKGKRGAKG<br>61- KQADVADQQTTELPAENGET<br>81- ENQSPASEEEKEAKSD
Human	HMGN1 (1-100 a.a.)	hH	1- MPKRKVSSAEGAAKEEPKRR<br>21- SARLSAKPPAKVEAKPKKAA<br>41- AKDKSSDKKVQTKGKRGAKG<br>61- KQAEVANQETKEDLPAENGE<br>81- TKTEESPASDEAGEKEAKSD

Table S2 │Antibodies for flow cytometry

Antibody	Clone	Antibody	Clone
CD3e	145-2C11	TIGIT	1G9
CD4	RM4-4	TIM-3	RMT3-23
CD8a	53-6.7	PD-1 / CD279	RMP1-30
CD11b	M1/70	Ly-6C	HK1.4
CD11c	N418	Ly-6G	1A8
CD28	37.51	CCR-7	4B12
CD44	IM7	IL-2	JES6-5H4
CD45	30-F11	IFN-γ	XMG1.2
B220	RA3-6B2	TNF-α	MP6-XT22
CD49b / Pan NK	DX5	**Isotype Controls**	
CD90.1	OX-7	Mouse IgG	MOPC-21
CD137	17B5	Rat IgG1	HRPN
ICOS / CD278	C398.4A	Rat IgG2b	LTF-2
LAG-3	eBioC9B7W	American Hamster IgG	HTK888

Monoclonal antibodies (mAbs) were purchased from BD bioscience, BioLegend, eBioscience or Tonbo bioscience.

Table S3 │Primers for quantitative real-time PCR

Name	Sequences (5' – 3')	Species	Marker
Cytotoxic T-lymphocyte-associated protein 4 (Ctla4), F	TCATGTACCCACCGCCATAC	mus musculus	Eurofins (JP)
Cytotoxic T-lymphocyte-associated protein 4 (Ctla4), R	CCCCAAGCTAACTGCGACAA	mus musculus	Eurofins (JP)
Cytotoxic T-lymphocyte-associated protein 4 (Ctla4), Probe	TTGTGGGCATGGGCAACGGGACGCAGA	mus musculus	Eurofins (JP)
Glyceraldehyde-3-phosphate dehydrogenase (Gapdh), F	AGTATGACTCCACTCACGGCAA	mus musculus	Sigma-Aldrich (USA)
Glyceraldehyde-3-phosphate dehydrogenase (Gapdh), R	TCTCGCTCCTGGAAGATGGT	mus musculus	Sigma-Aldrich (USA)
Lymphocyte-activation gene 3 (Lag3), F	CACCTGTAGCATCCATCTGC	mus musculus	Eurofins (JP)
Lymphocyte-activation gene 3 (Lag3), R	CCAGGTAACCCGAAGGATTT	mus musculus	Eurofins (JP)
Programmed cell death 1 (Pdcd1), F	TGCAGTTGAGCTGGCAAT	mus musculus	Eurofins (JP)
Programmed cell death 1(Pdcd1), R	GGCTGGGTAGAAGGTGAGG	mus musculus	Eurofins (JP)
TIM-3 (Havcr2), F	TTTTCAGGTCTTACCCTCAACTG	mus musculus	Eurofins (JP)
TIM-3 (Havcr2), R	CATAAGCATTTTCCAATGACCTT	mus musculus	Eurofins (JP)

Table S4 │Gene ontology (GO) analysis of GO: biological processes and KEGG pathway

ID	GO term (upregulated)	Term *p* value	Nr. genes
KEGG:03010	Ribosome	4.95E-44	36.00
GO:0006412	translation	5.49E-25	37.00
GO:0043043	peptide biosynthetic process	1.64E-24	37.00
GO:0043604	amide biosynthetic process	6.51E-23	37.00
GO:0006518	peptide metabolic process	9.93E-22	37.00
GO:0043603	cellular amide metabolic process	1.93E-19	37.00
GO:1901566	organonitrogen compound biosynthetic process	1.44E-17	40.00
GO:0000028	ribosomal small subunit assembly	8.26E-10	6.00
GO:0042274	ribosomal small subunit biogenesis	1.47E-08	8.00
GO:0042254	ribosome biogenesis	2.07E-08	13.00
GO:0022613	ribonucleoprotein complex biogenesis	6.30E-08	15.00
GO:0042255	ribosome assembly	1.28E-07	7.00
GO:2000341	regulation of chemokine (C-X-C motif) ligand 2 production	3.59E-07	4.00
GO:0034341	response to interferon-gamma	4.20E-07	8.00
GO:0006123	mitochondrial electron transport, cytochrome c to oxygen	8.37E-07	4.00
GO:0072567	chemokine (C-X-C motif) ligand 2 production	8.37E-07	4.00
GO:0022618	ribonucleoprotein complex assembly	8.53E-06	9.00
GO:2000343	positive regulation of chemokine (C-X-C motif) ligand 2 production	9.43E-06	3.00
GO:0071826	ribonucleoprotein complex subunit organization	1.35E-05	9.00
GO:0042775	mitochondrial ATP synthesis coupled electron transport	2.27E-05	5.00
GO:0006364	rRNA processing	3.05E-05	8.00
GO:0042773	ATP synthesis coupled electron transport	3.27E-05	5.00
GO:0046596	regulation of viral entry into host cell	3.68E-05	4.00
GO:1902253	regulation of intrinsic apoptotic signaling pathway by p53 class mediator	4.23E-05	4.00
KEGG:05016	Huntington's disease	4.28E-05	8.00
KEGG:04932	Non-alcoholic fatty liver disease (NAFLD)	6.25E-05	7.00
GO:0032642	regulation of chemokine production	1.03E-04	5.00
GO:0022904	respiratory electron transport chain	1.43E-04	5.00
GO:0032602	chemokine production	1.43E-04	5.00
GO:0034643	establishment of mitochondrion localization, microtubule-mediated	1.45E-04	3.00
GO:0047497	mitochondrion transport along microtubule	1.45E-04	3.00
GO:0006119	oxidative phosphorylation	1.82E-04	5.00
GO:0046597	negative regulation of viral entry into host cell	2.09E-04	3.00
GO:0045624	positive regulation of T-helper cell differentiation	2.09E-04	3.00
GO:0045071	negative regulation of viral genome replication	2.13E-04	4.00
GO:0022900	electron transport chain	2.28E-04	5.00
GO:0051654	establishment of mitochondrion localization	2.46E-04	3.00

GO:0002699	positive regulation of immune effector process	2.52E-04	7.00
KEGG:00190	Oxidative phosphorylation	2.56E-04	6.00
GO:0072332	intrinsic apoptotic signaling pathway by p53 class mediator	2.69E-04	5.00
GO:0035456	response to interferon-beta	2.74E-04	4.00
GO:0030490	maturation of SSU-rRNA	3.46E-04	4.00
GO:0032722	positive regulation of chemokine production	3.46E-04	4.00
GO:0035455	response to interferon-alpha	3.86E-04	3.00
GO:0002293	alpha-beta T cell differentiation involved in immune response	4.02E-04	4.00
GO:0022408	negative regulation of cell-cell adhesion	4.05E-04	6.00
GO:0046632	alpha-beta T cell differentiation	4.25E-04	5.00
GO:0002287	alpha-beta T cell activation involved in immune response	4.32E-04	4.00
GO:0002286	T cell activation involved in immune response	5.12E-04	5.00

ID	GO term (downregulated)	Term *p* value	Nr. genes
GO:0006996	organelle organization	4.71E-18	124.00
GO:1903047	mitotic cell cycle process	1.67E-14	45.00
GO:0051276	chromosome organization	6.70E-14	55.00
GO:0022402	cell cycle process	1.02E-13	55.00
GO:0007049	cell cycle	1.77E-13	66.00
GO:0000280	nuclear division	1.13E-12	38.00
GO:0051301	cell division	1.14E-12	37.00
GO:0000278	mitotic cell cycle	1.35E-12	45.00
GO:0007067	mitotic nuclear division	3.09E-12	32.00
GO:0048285	organelle fission	1.00E-11	38.00
GO:0006259	DNA metabolic process	4.23E-11	44.00
GO:0051052	regulation of DNA metabolic process	9.86E-10	26.00
GO:0007059	chromosome segregation	5.82E-09	22.00
KEGG:04110	Cell cycle	1.79E-08	14.00
GO:0051172	negative regulation of nitrogen compound metabolic process	2.21E-08	57.00
GO:0045934	negative regulation of nucleobase-containing compound metabolic process	3.61E-08	53.00
GO:0000723	telomere maintenance	6.36E-08	13.00
GO:0032200	telomere organization	7.05E-08	13.00
GO:0006261	DNA-dependent DNA replication	7.81E-08	13.00
GO:0007051	spindle organization	1.11E-07	14.00
GO:0006260	DNA replication	1.38E-07	18.00
GO:0010558	negative regulation of macromolecule biosynthetic process	1.64E-07	52.00
GO:2000113	negative regulation of cellular macromolecule biosynthetic process	1.69E-07	50.00
GO:0010605	negative regulation of macromolecule metabolic process	1.99E-07	74.00
GO:0009890	negative regulation of biosynthetic process	2.11E-07	54.00
GO:0006281	DNA repair	2.41E-07	24.00

GO:0000070	mitotic sister chromatid segregation	2.46E-07	13.00
GO:0031327	negative regulation of cellular biosynthetic process	3.07E-07	53.00
GO:0031324	negative regulation of cellular metabolic process	3.13E-07	74.00
GO:0000226	microtubule cytoskeleton organization	4.19E-07	25.00
GO:0060249	anatomical structure homeostasis	4.33E-07	21.00
GO:0000819	sister chromatid segregation	4.45E-07	14.00
GO:0098813	nuclear chromosome segregation	4.87E-07	17.00
GO:0033044	regulation of chromosome organization	6.25E-07	19.00
GO:0033554	cellular response to stress	6.92E-07	54.00
GO:0071103	DNA conformation change	1.81E-06	15.00
GO:0051726	regulation of cell cycle	1.89E-06	35.00
GO:0071897	DNA biosynthetic process	2.64E-06	13.00
GO:0006302	double-strand break repair	2.71E-06	14.00
GO:0051054	positive regulation of DNA metabolic process	2.90E-06	15.00
GO:0051173	positive regulation of nitrogen compound metabolic process	3.11E-06	57.00
GO:0032210	regulation of telomere maintenance via telomerase	6.02E-06	7.00
GO:0051649	establishment of localization in cell	7.68E-06	54.00
GO:0044772	mitotic cell cycle phase transition	9.60E-06	18.00
GO:0046907	intracellular transport	1.08E-05	46.00
GO:0006974	cellular response to DNA damage stimulus	1.11E-05	28.00
GO:1902850	microtubule cytoskeleton organization involved in mitosis	1.34E-05	9.00
GO:0007052	mitotic spindle organization	1.34E-05	9.00
GO:0010564	regulation of cell cycle process	1.37E-05	23.00
GO:0051253	negative regulation of RNA metabolic process	1.39E-05	43.00
GO:0007346	regulation of mitotic cell cycle	1.49E-05	21.00
GO:0007010	cytoskeleton organization	1.53E-05	40.00
GO:1902679	negative regulation of RNA biosynthetic process	1.61E-05	42.00
GO:0010833	telomere maintenance via telomere lengthening	1.75E-05	8.00
GO:0034502	protein localization to chromosome	1.95E-05	8.00
GO:0007004	telomere maintenance via telomerase	2.20E-05	7.00
GO:0033043	regulation of organelle organization	2.28E-05	39.00
GO:1903507	negative regulation of nucleic acid-templated transcription	2.46E-05	41.00
GO:0090307	mitotic spindle assembly	2.50E-05	7.00
GO:1904356	regulation of telomere maintenance via telomere lengthening	2.50E-05	7.00
GO:0032204	regulation of telomere maintenance	2.66E-05	8.00
GO:0045935	positive regulation of nucleobase-containing compound metabolic process	2.72E-05	52.00
GO:0044770	cell cycle phase transition	2.76E-05	18.00
GO:0045892	negative regulation of transcription, DNA-templated	3.29E-05	40.00
